# Landscape connectivity and climatic drivers shaping Noctuidae communities in a Brazilian seasonally dryland fruit-producing region

**DOI:** 10.1007/s13744-026-01446-y

**Published:** 2026-10-28

**Authors:** Tiago Cardoso Costa-Lima, José Bruno Malaquias, Angélica da Silva Salustino, Magali Haidée Pereira Martínez, Alexandre Specht

**Affiliations:** 1 Embrapa Semi-Arid Region, Petrolina, Pernambuco 56302-970 Brazil; 2 Federal University of Paraíba (UFPB), Areia, Paraíba Brazil; 3 Embrapa Genetic Resources & Biotechnology, Brasília, Distrito Federal Brazil

**Keywords:** *Spodoptera*, *Elaphria*, grapes, light trap, wind speed, precipitation

## Abstract

Drylands are highly sensitive to climatic shifts, affecting insect population dynamics and multi-trophic interactions. This study evaluated Noctuidae diversity and temporal distribution in the São Francisco Valley, a semi-arid, irrigated, fruit-producing region in Northeast Brazil, in relation to local environmental factors. We conducted monitoring over two consecutive years using Pennsylvania light traps deployed across two agricultural landscapes integrated with native Caatinga dry forest: an experimental multi-crop station and a commercial table grape farm. We captured 12,407 Noctuidae moths; raw richness favored the grapevine site, but diversity did not differ, and per-individual richness favored the multi-crop site once sampling effort was considered*.* Four Noctuinae species dominated the assemblage (94.04% of total captures): *Spodoptera frugiperda* (Smith) (39.53%), *Elaphria agrotina* (Guenée) (36.89%), *Spodoptera cosmioides* (Walker) (9.21%), and *Spodoptera albula* (Walker) (8.40%). Immediate captures of *S. frugiperda* and *S. albula* rose with lower wind speed and higher precipitation; previous-month captures showed the same patterns for *S. frugiperda* and *S. albula*, but *E. agrotina* responded to temperature and *S. cosmioides* to site and precipitation instead. Circular statistics indicated a stark temporal segregation between *S. frugiperda* and *S. cosmioides*. These findings suggest Caatinga connectivity elevates vineyard abundance, whereas the multi-crop mosaic’s heterogeneity yields higher per-individual richness*.* Specialized crop management practices, such as continuous vineyard pruning, generate stable niches that favor detritivorous species like *E. agrotina*. Local multi-year calibrations are essential for accurate pest forecasting and integrated management across agricultural-forest interfaces in semi-arid zones.

**Supplementary Information:**

The online version contains supplementary material available at https://doi.org/10.1007/s13744-026-01446-y.

## Introduction

Drylands are areas with a low ratio of mean annual precipitation to potential evapotranspiration (aridity index ranging from 0.05 to 0.65) (Lal 2004) and cover around 45% of the Earth’s surface (Prăvălie 2016). These areas become more sensitive to climatic changes, including increases in temperature, aridity, and precipitation (Ahmed et al. 2022; Naorem 2023). Rainfall and its temporal distribution can shape the species communities and multi-trophic interactions in arid and semi-arid environments (Fischer et al. 2022). Under climate change, insect monitoring is fundamental to detecting changes in species composition and activity patterns (Eickermann et al. 2023).

Noctuidae is the third-largest family of Lepidoptera, with more than 1000 genera and almost 12,000 species (Van Nieukerken et al. 2011). This moth family is one of the most ecologically successful and economically important groups of organisms (Keegan et al. 2021). Global agricultural pests include the fall armyworm, *Spodoptera frugiperda* (Smith) (Tay et al. 2023), and the cotton bollworm, *Helicoverpa armigera* (Hübner) (Riaz et al. 2021). Population dynamics studies can predict species’ responses to climatic phenomena (Specht et al. 2022). For example, a study in the Brazilian Savannah found that precipitation well above or below the climatic norm reduced populations of three Noctuidae species, including *S. frugiperda* (Fonseca-Medrano et al. 2019). In the same region, dry seasons correlated with higher cutworm abundance in the genera *Feltia* and *Agrotis* (Specht et al. 2022). Noctuidae species are mainly identified from adult morphology. Moths are primarily crepuscular or nocturnal (Hahn and Brühl 2016). Thus, light trapping is especially indicated for this group (Macgregor et al. 2019), and it is one of the most common and efficient methods for sampling nocturnal flying insects and obtaining many clades and individuals (Montgomery et al. 2021).

Many Noctuidae species are fruit pests in Brazil. In grapevines, reports describe *S. frugiperda* causing defoliation (Bortolotto et al. 2021), and *Oraesia argyrosema* (Hampson) and *Gonodonta biarmata* Guenée can pierce the rind and pulp of grape berries (Zenker et al. 2010). In citrus, *H. armigera* was reported to cause defoliation and feeding on fruits (Bueno et al. 2014). *Chrysodeixis includens* (Walker) was observed to cause defoliation in passion flower plants (Benassi et al. 2012), and *Chloridea (Heliothis) subflexa* (Guenée) was reported to damage cape gooseberry fruits (de Melo et al. 2017).

Brazil’s most important tropical fruit-producing region is the São Francisco Valley, a dryland area in northeast Brazil. Table grapes and mangoes are exported to many countries, while other fruits, such as coconut, guava, acerola, and others, are produced for internal commerce (Telhado and Capdeville 2021). Agriculture in the region is possible through irrigation. The annual rainfall average is 567 mm, with over 90% concentrated from November to April (Embrapa 2026). Knowledge of Noctuidae species in the region is limited, and no studies have examined their temporal distribution. Therefore, this study aimed to (i) characterize the composition and diversity of Noctuidae assemblages in two contrasting agricultural landscapes embedded in the Caatinga dry forest; (ii) determine the relative influence of local climatic variables on the abundance of the dominant species, considering both immediate and one-month-lagged effects; and (iii) assess whether the dominant species exhibit temporal partitioning of their seasonal peaks. We hypothesized that, because of differences in landscape connectivity and crop management, the two habitats would differ in community composition and dominance structure, and that dominant species would show climate-linked, temporally segregated population peaks that reduce competitive overlap.

## Material and methods

### Study area

**Fig. 1:**
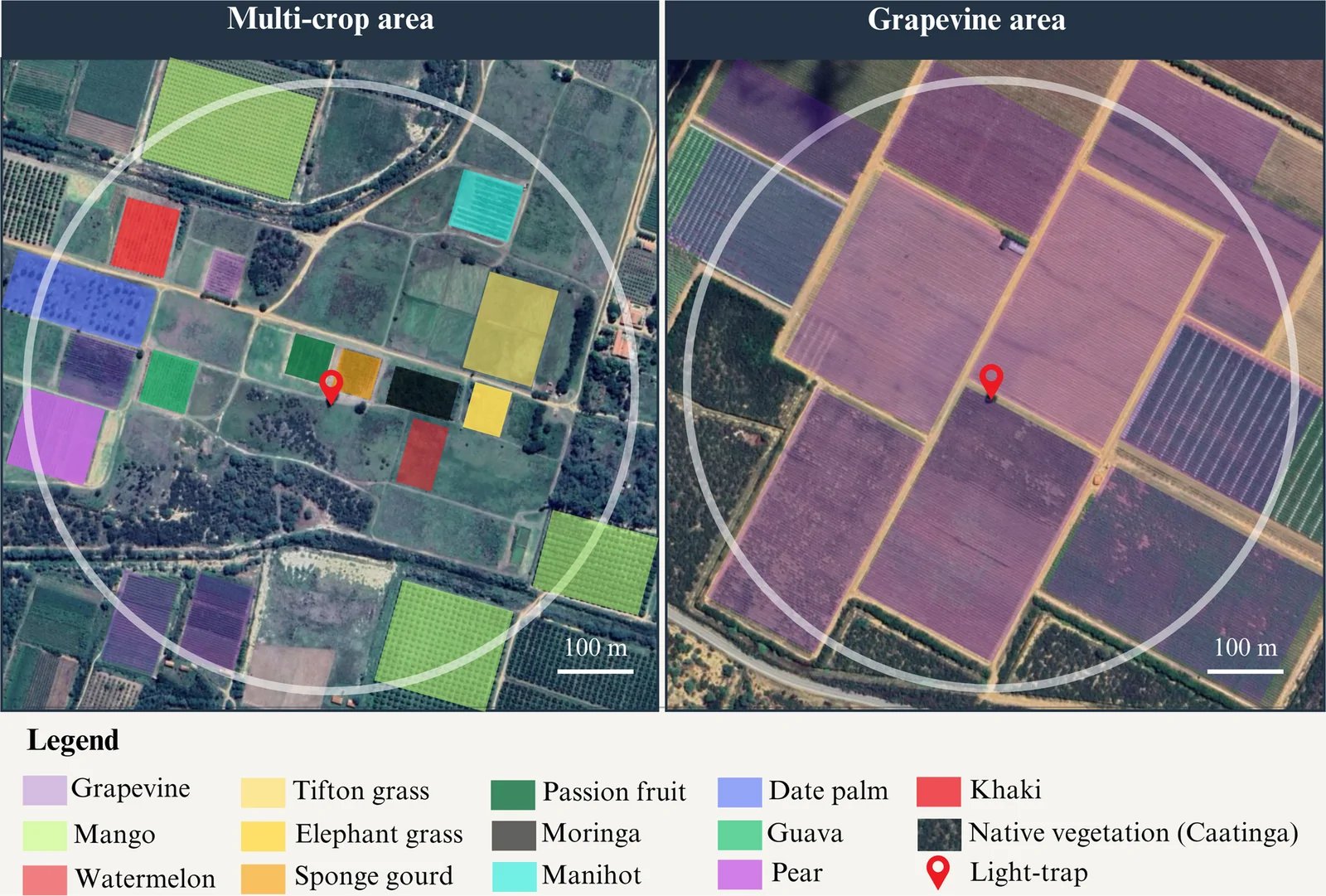
Map of the multi-crop and grapevine area used in the study, showing the crops within a 400-m radius (circle) around the light trap, São Francisco Valley, Brazil (Google Earth image)

We conducted Noctuidae monitoring from July 2015 to June 2017 in two areas of the São Francisco Valley, Brazil. The semi-arid regional climate is classified as BSh according to Köppen (Alvares et al. 2013). The first study site was at an Embrapa research station in Petrolina (09°08′14.4″S; 40°18′7.4″W). Within a 400-m radius from the trap, there were experimental plantations of watermelon, tifton grass, elephant grass, mango, manihot, grapevine, date palm, sponge gourd (*Luffa aegyptiaca* Mill.), moringa (*Moringa oleifera* Lam.), passion fruit, guava, pear, khaki, and native plants from the Caatinga, a tropical dry forest found only in Brazil (Antongiovanni et al. 2018). The second was on a table grape farm covering 338 ha in Lagoa Grande (09°03′54.3″S; 40°10′23.6″W). Within a 400-m radius of the trap, grape vineyards and native Caatinga plants were present (Fig. 1).

### Moth sampling and identification

We assessed Noctuidae diversity in both areas over 2 years by sampling moths with Pennsylvania light traps (Frost 1957). The traps were equipped with a BL T8 15 W black fluorescent light, which was activated for five consecutive nights during each *novilunium* to reduce the effect of the moonlight. Each day, we separated the collected insects and preserved them in 96% (v/v) ethyl alcohol for future sorting and specific identification. We installed each trap 3 m above the ground. For statistical purposes, we treated each night as a replicate (Fonseca-Medrano et al. 2019). Family-level identification followed the Lepidoptera identification keys (Carneiro et al. 2024). We identified subfamilies and species using specific literature, including Lafontaine and Poole (1991); Poole et al. (1993); Pogue (2002, 2004); Lafontaine (2004); and Madruga et al. (2022). We accessed the Embrapa Semiarid insect collection to check for host-plant records in the SFV for species sampled in the study.

### Climatic and meteorological variables

We used stations present in both study areas (Embrapa Research Station and the table grape farm) to assess meteorological data. Selected variables included temperature (minimum, average, and maximum), relative air humidity (minimum, average, and maximum), global solar radiation, wind speed, precipitation, and evapotranspiration.

### Data analysis

We calculated faunistic parameters using abundance data to evaluate and compare moth community structure between the grapevine and multi-crop sampling sites. For each sampling unit (daily trap catch), we calculated three ecological indices: species richness (*S*), the Shannon–Wiener diversity index (*H*’), and Pielou’s evenness index (*J*’). These community metrics were computed using the *vegan* package in the R statistical environment (R Core Team 2026). To assess significant differences in diversity indices between the two agricultural habitats, we fitted mixed-effects models with a random intercept for sampling occasion (site × novilunium trapping bout), because the five consecutive nightly samples within each trapping bout are not statistically independent. Species richness was modeled with a negative-binomial generalized linear mixed model (GLMM), Shannon diversity with a Gaussian linear mixed model (LMM), and Pielou’s evenness with a beta GLMM. Because realized sampling effort (individuals captured; the trapping protocol was identical between sites) differed substantially between sites (9008 individuals at the grapevine site vs. 3399 at the multi-crop site over the two years), we additionally refit these models including the number of individuals captured on each trapping night as an effort term (an offset for richness; a covariate for Shannon diversity and evenness), cross-validating the effort-adjusted richness model across three independent implementations (glmmTMB, lme4::glmer.nb and MASS::glm.nb). As a complementary, model-free check, the pooled 2-year abundance totals were also standardized to equal sampling effort using individual-based rarefaction (2000 resampling replicates) and Chao1 richness estimation. Table 1 summarizes the three comparison levels: effort-unadjusted, pooled-effort-standardized, and nightly effort-adjusted*.* Data visualization was performed using the *ggplot2*, *ggdist*, and *patchwork* packages. All statistical analyses and graphical representations were conducted in the R statistical program (R Core Team 2026).

**Table 1:** Site effect on species richness (S), Shannon diversity (H’), and Pielou’s evenness (J’) under three levels of effort correction: pseudoreplication-only (Fig. 2), rarefaction of the pooled 2-year totals (2000 replicates), and nightly effort-adjustment (nightly capture number as an offset for S and covariate for H’/J’; random intercept for sampling occasion; cross-validated with glmmTMB, lme4::glmer.nb and MASS::glm.nb). *n.s.*, not significant (*p* > 0.05)

Index	No effort correction	Pooled 2-year total, rarefied to equal N (empirical p)	Nightly model, adjusted for nightly effort (offset/covariate)
Richness (S)	z = 5.01, *p* < 0.001 (grapevine higher)	*p* = 0.385 (n.s.)	*z* = 2.90, *p* = 0.004 (multi-crop higher)
Shannon (H’)	*t*₄₆ = 2.96, *p* = 0.005 (grapevine higher)	*p* = 0.745 (n.s.)	*t*₄₇ = 0.24, *p* = 0.813 (n.s.)
Evenness (J’)	z = 2.64, *p* = 0.008 (multi-crop higher)	*p* = 0.165 (n.s.)	*z* = 0.34, *p* = 0.734 (n.s.)

To identify the influence of climatological variables on the monthly captures of the four most abundant species, we analyzed concurrent-month and one-month-lagged (lag-1) climatic variables in parallel. For each time window, we pooled the ten standardized monthly climatic variables across both sites and reduced them to a small number of orthogonal axes using principal component analysis (PCA; prcomp function, *stats* package), which addressed multicollinearity among the raw variables. The concurrent-month analysis used *n* = 48 site months (24 months × 2 sites) and the lag-1 analysis used *n* = 46 occasions (excluding the first sampled month at each site, which has no preceding month). Biplots were constructed with *ggplot2* and enhanced with *ggrepel*. Monthly abundance of each of the four dominant species was then modeled as a function of PC1, PC2, and PC3, site, and their interactions, using negative binomial generalized linear mixed models.

A circular statistics analysis was conducted to evaluate temporal directionality (macro-seasonality) and identify the degree of population synchrony among the four most abundant species [*Elaphria agrotina* (Guenée), *Spodoptera cosmioides* (Walker), *S. frugiperda*, and *Spodoptera albula* (Walker)] throughout the calendar year. Occurrence dates were transformed into radial angles distributed across 360°, allowing for the calculation of the mean vector direction (μ), circular standard error, 95% confidence intervals based on the von Mises distribution, and the mean vector length (*ρ*) to estimate the seasonal concentration of insects across four distinct cropping cycles (grapevine and multi-crop areas evaluated over two consecutive years). We used individual observations (N) for each species across all sampled nights to estimate these circular parameters, and applied Rayleigh tests to assess circular uniformity. Subsequently, a circular correlation test (cor.circular) was applied using the estimated mean direction vectors from the four cropping cycles (*n* = 4 macro-environmental cycles) to evaluate interspecific temporal association (synchrony, positive values; or seasonal segregation, negative values) between species peaks. Mean vectors, confidence intervals, *ρ*, and Rayleigh test results are additionally reported separately for each of the four independent site × year cropping cycles, and pooled per species, in Tables S5 and S6—Supplementary Information. We performed all statistical analyses and generated plots using R (R Core Team 2026).

### AI-powered tool disclosure

The AI-powered academic search engine Consensus (Consensus App Inc.) was utilized during the literature review process to assist in identifying relevant peer-reviewed studies. AI assistant Claude (Anthropic PBC) was used to assist with language revision of the manuscript text and with the implementation of R code for the statistical analyses. The authors independently verified and validated all retrieved literature, data interpretations, statistical code, and final text edits to ensure accuracy, originality, and scientific rigor.

## Results

### Composition of the noctuidae assemblage

Over 2 years, 12,407 Noctuidae moths, representing six subfamilies (Bagisarinae, Eriopinae, Heliothinae, Noctuinae, Oncocnemidinae, and Plusiinae), were captured, with 3399 in the multi-crop area and 9008 in the grapevine area. The majority of the specimens were identified (99.68%). The few specimens that could not be identified were completely descaled or had body parts and wings fragmented by other insects, especially coleopterans, which have a more chitinized body. The Noctuinae subfamily was the most frequent and abundant (97.73%), with four species representing 94.04% of the total captured moths: *S. frugiperda* (39.53%), *E. agrotina* (36.89%), *S. cosmioides* (9.21%), and *S. albula* (8.40%). The other 20 species had frequencies below 2.2% (Table S1—Supplementary Information).

### Faunistic analysis

**Fig. 2:**
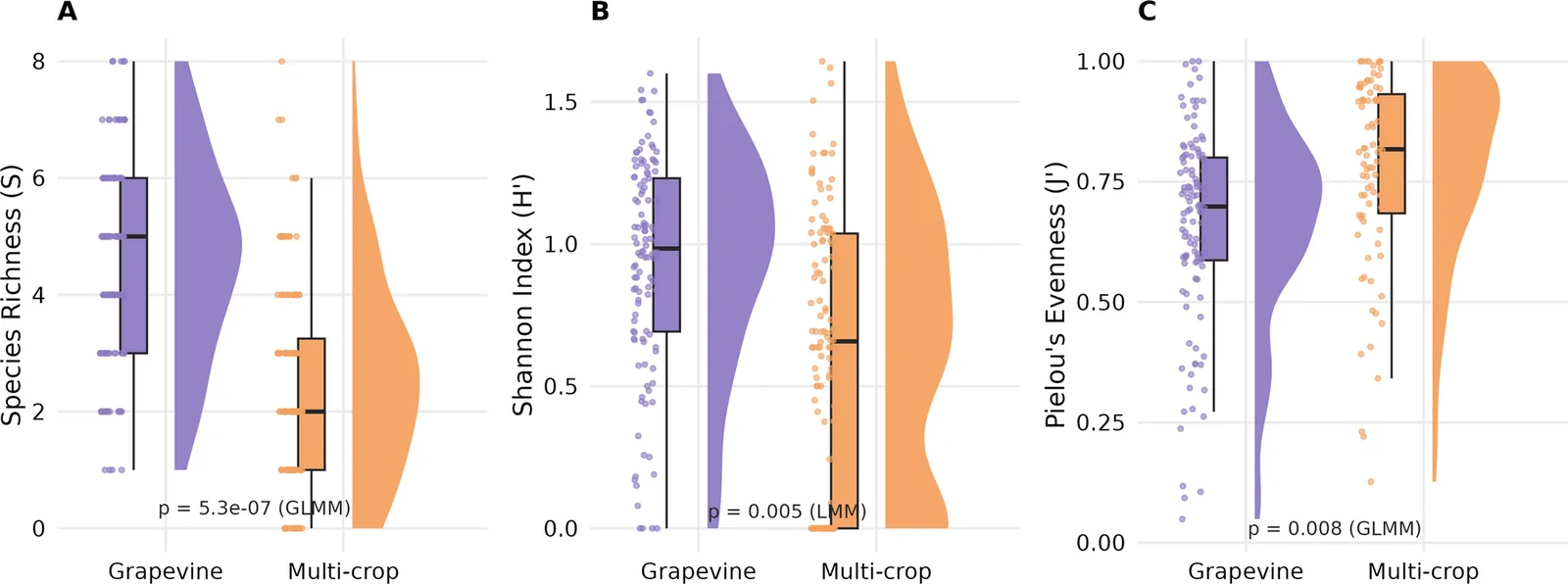
Ecological diversity of Noctuidae moth communities based on a two-year survey (July 2015–June 2017) conducted in two areas (grapevine and multi-crop) of a seasonally dryland fruit-producing region, São Francisco Valley, Brazil. Panels show **A** species richness, **B** Shannon–Wiener diversity, and **C** Pielou’s Evenness per sampling night (*n* = 120 nights per site). Raincloud plots display data density, boxplots (median and interquartile range), and individual nightly observations (points). *p*-values are from mixed-effects models with a random intercept for sampling occasion (negative-binomial GLMM for S; beta GLMM for J’; Gaussian LMM for H’). These models correct for temporal pseudoreplication among nights but not for the unequal total sampling effort (individuals captured) between sites; see Table 1 for comparisons standardized to equal sampling effort, which show a different pattern for all three indices

**Fig. 3:**
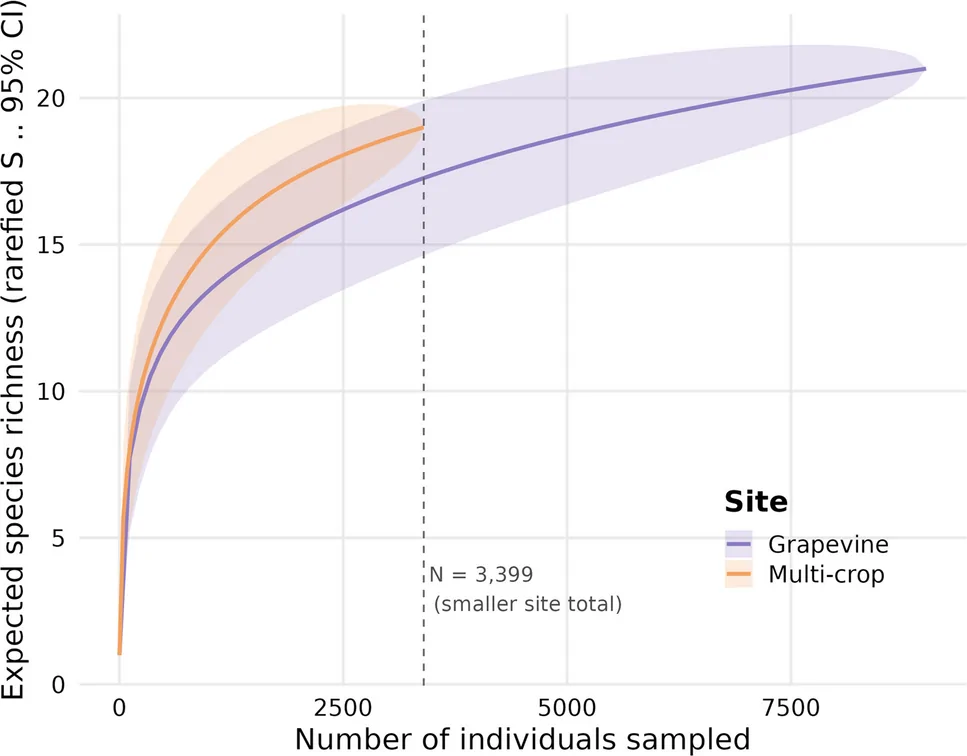
Individual-based rarefaction curves (expected species richness ± 95% CI) for the grapevine and multi-crop sites, based on the pooled 2-year abundance data. The dashed vertical line marks the total number of individuals collected at the smaller site (multi-crop, *N* = 3399); at this common sampling effort, expected richness is statistically indistinguishable between habitats

Realized sampling effort, the number of individuals captured, the standard currency in individual-based rarefaction (Gotelli and Colwell 2001), differed substantially between sites despite an identical trapping protocol (9008 individuals recorded at the grapevine site versus 3399 at the multi-crop site). We therefore evaluated whether the observed diversity patterns reflect genuine habitat differences or simply the far greater number of individuals captured at the grapevine site. Because the five consecutive trapping nights within each novilunium bout are not statistically independent, all comparisons below use mixed-effects models with a random intercept for sampling occasion. At the observed (unequal) effort, these models indicated higher richness and Shannon diversity at the grapevine site (Fig. 2; negative-binomial GLMM, z = 5.01, *p* < 0.001; Gaussian LMM, t₄₆ = 2.96, *p* = 0.005) and higher evenness at the multi-crop site (beta GLMM; z = 2.64, *p* = 0.008). However, once sampling effort was standardized, using individual-based rarefaction of the pooled 2-year totals (Fig. 3; Table S2—Supplementary Information) and, more powerfully, models that additionally adjust for the number of individuals captured on each trapping night (Table 1), Shannon diversity and evenness no longer differed between sites (all *p* > 0.16), and species richness reversed direction, becoming higher at the multi-crop site once effort was accounted for (*z* = 2.90, *p* = 0.004). These results indicate that the raw diversity contrast between sites (Fig. 2) is driven largely by unequal numbers of individuals captured rather than by an underlying ecological difference, and that, if anything, the multi-crop community is proportionally richer in species per individual captured.

### Influence of climatic factors

**Fig. 4:**
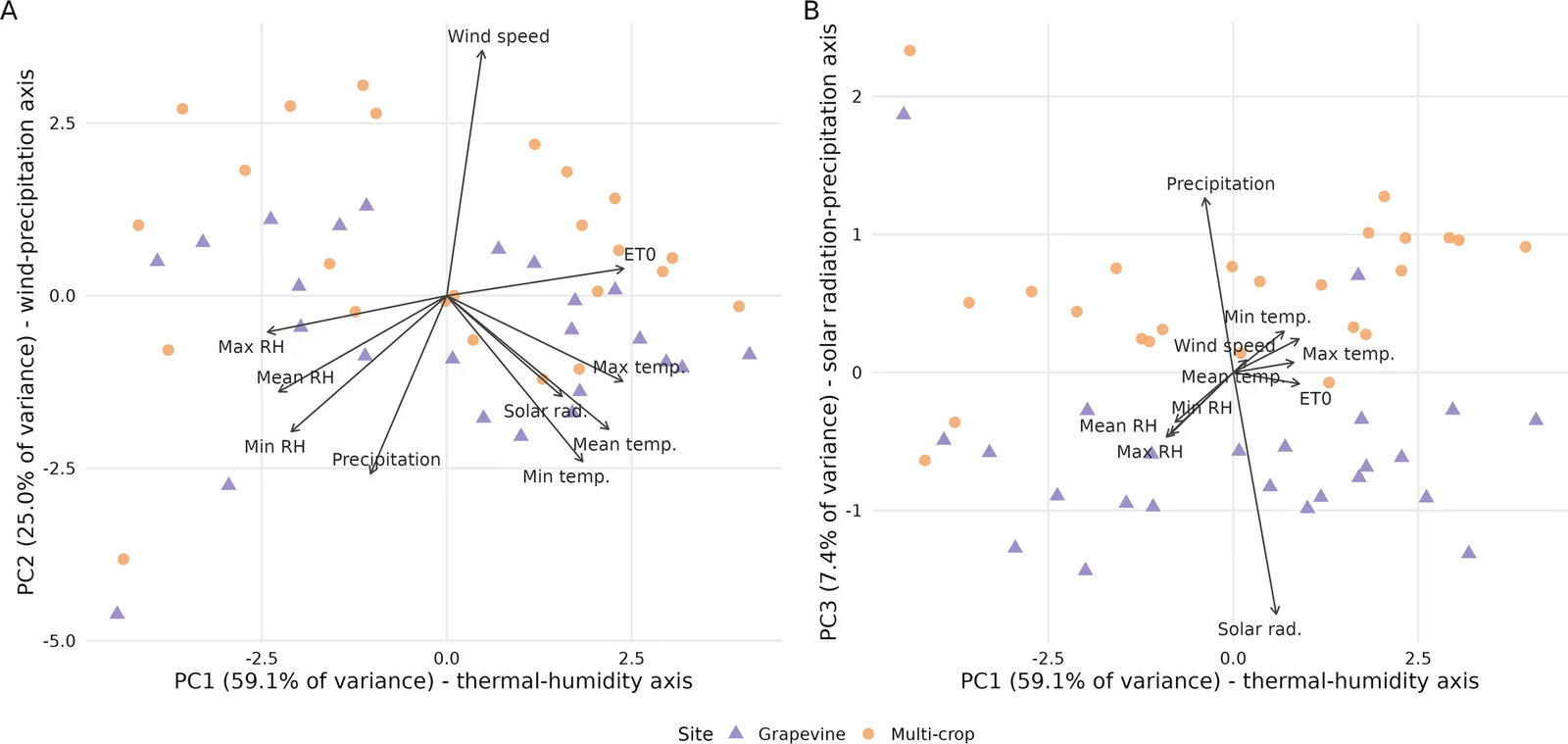
Principal component analysis of ten standardized monthly climatic variables, pooling both the grapevine and multi-crop sites (*n* = 24 months × 2 sites = 48 occasions). **A** PC1 (59.1% of variance) contrasts higher temperature and evapotranspiration against higher relative humidity. PC2 (25.0%) contrasts higher wind speed against higher precipitation and higher minimum temperature. **B** PC3 (7.4%) contrasts higher solar radiation against higher precipitation, plotted against PC1. Arrows show variable loadings; points show monthly climate at each site

Analyzing climatic data from the same month as noctuid captures, the first three principal components accounted for 91.5% of the variability in ten standardized climatic variables pooled across both sites (59.1%, 25.0%, and 7.4% for PC1, PC2, and PC3, respectively; Fig. 4, Table S3—Supplementary Information)*.* PC1 contrasted higher temperature, evapotranspiration, and lower humidity with lower temperature and higher humidity; PC2 contrasted higher wind speed with higher precipitation and lower minimum temperature; and PC3 contrasted higher solar radiation with higher precipitation, with the two loadings in opposite directions. Monthly abundance of the four dominant species, modeled as a function of PC1–PC3, site, and their interactions (negative-binomial GLMMs; Table 2), showed distinct climatic responses. *Spodoptera frugiperda* was more abundant at higher temperature (PC1, *β* = 0.47, *p* < 0.001) and at lower wind speed/precipitation (PC2, *β* = − 0.80, *p* < 0.001), with this response differing between sites (PC2 × site, *β* = 0.58, *p* = 0.031). *Spodoptera albula* was more abundant at lower wind speed/precipitation (PC2, *β* = − 1.03, *p* < 0.001) and higher solar radiation (PC3, *β* = − 2.24, *p* = 0.049), and less abundant at the grapevine site than the multi-crop site (*β* = − 2.23, *p* = 0.015). *Spodoptera cosmioides* showed no response to temperature, wind speed/precipitation, or solar radiation alone (all *p* > 0.085), but was more abundant at the grapevine site (*β* = 1.77, *p* = 0.020), with this response differing between sites (PC2 × site, *β* = 0.95, *p* = 0.007). *Elaphria agrotina* was more abundant under higher solar radiation alone (PC3, *β* = − 2.67, p = 0.003), with no response to temperature, wind speed/precipitation, or site (Table 2).

**Table 2:** Coefficients (log scale) from negative-binomial GLMMs of monthly abundance for the four dominant species, as a function of the concurrent-month climatic PCA axes (PC1–PC3), site, and their interactions. n.s. = *p* > 0.05

Species	PC1 (hot–dry axis)	PC2 (wind/precip. axis)	PC3	Site (multi-crop → grapevine)	PC2 × site
*S. frugiperda*	+ 0.47, *p* < 0.001	− 0.80, *p* < 0.001	n.s	n.s	+ 0.58, *p* = 0.031
*S. albula*	n.s	− 1.03, *p* < 0.001	− 2.24, *p* = 0.049	− 2.23, *p* = 0.015	n.s
*S. cosmioides*	n.s	n.s. (*p* = 0.085)	n.s. (*p* = 0.060)	+ 1.77, *p* = 0.020	+ 0.95, *p* = 0.007
*E. agrotina*	n.s	n.s	− 2.67, *p* = 0.003	n.s	n.s

**Fig. 5:**
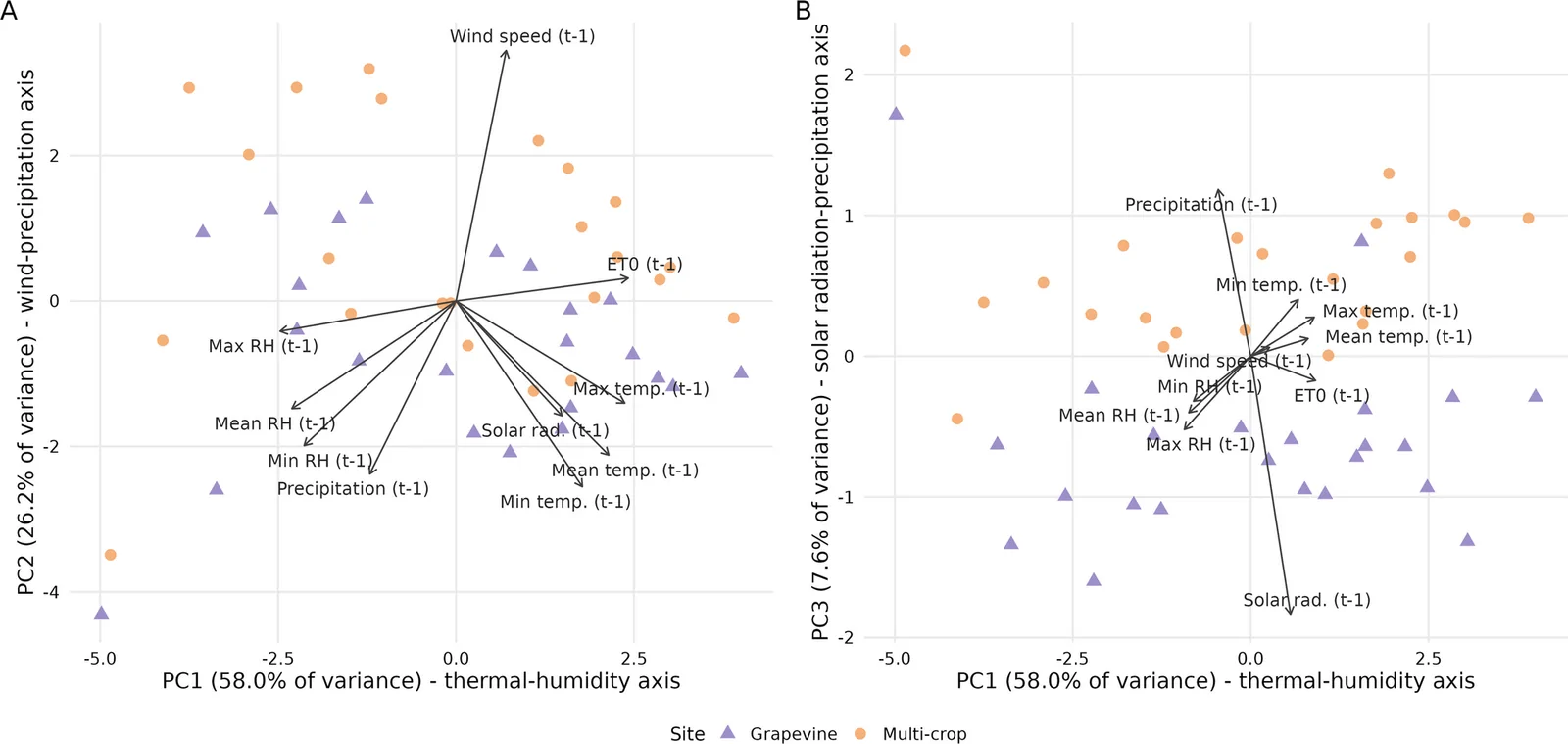
Principal component analysis of ten standardized 1-month-lagged (lag-1) monthly climatic variables, pooling both the grapevine and multi-crop sites (*n* = 46 occasions). **A** PC1 (58.0% of variance) contrasts higher temperature and evapotranspiration with higher relative humidity. PC2 (26.2%) contrasts higher wind speed against higher precipitation and higher minimum temperature. **B** PC3 (7.6%) contrasts higher solar radiation against higher precipitation, plotted against PC1. Arrows show variable loadings; points show the preceding month’s climate at each site

Considering the climatic data from the month preceding noctuid captures, the first three principal components accounted for 91.7% of the variability in ten standardized lagged climatic variables pooled across both sites (58.0%, 26.2%, and 7.6% for PC1, PC2, and PC3, respectively; Fig. 5, Table S4—Supplementary Information), reflecting the same temperature (PC1), wind speed/precipitation (PC2), and solar radiation (PC3) axes described above for the concurrent month. Monthly abundance of the four dominant species, modeled as a function of these three lagged axes, site, and their interactions (negative-binomial GLMMs; Table 3), showed climatic responses broadly consistent with, but not identical to, the concurrent-month results. *Spodoptera frugiperda* was more abundant at higher preceding-month temperature (PC1, *β* = 0.29, *p* < 0.001), lower wind speed and higher precipitation (PC2, *β* = − 0.79, *p* < 0.001), and higher solar radiation (PC3, *β* = − 1.55, *p* < 0.001), with both responses differing between sites (PC2 × site, *β* = 0.77, *p* < 0.001; PC3 × site, *β* = 2.01, *p* < 0.001). *Spodoptera albula* was more abundant at lower wind speed and higher precipitation (PC2, *β* = − 0.83, *p* < 0.001) and under higher solar radiation (PC3, *β* = − 2.73, *p* < 0.001), and was less abundant overall at the grapevine site than at the multi-crop site (*β* = − 3.25, *p* < 0.001), with this response also differing between sites (PC1 × site, *β* = − 0.49, *p* = 0.019). *Spodoptera cosmioides* was more abundant at lower wind speed and higher precipitation (PC2, *β* = − 0.56, *p* = 0.010) and overall at the grapevine site (*β* = 2.77, *p* < 0.001), with this response also differing between sites (PC2 × site, *β* = 1.08, *p* < 0.001). *Elaphria agrotina* was more abundant at higher preceding-month temperature alone (PC1, *β* = 0.56, *p* = 0.017), with no detectable response to wind speed/precipitation, solar radiation, or site (Table 3).

**Table 3:** Coefficients (log scale) from negative-binomial GLMMs of monthly abundance for the four dominant species, as a function of the one-month-lagged (lag-1) climatic PCA axes (PC1–PC3), site, and their interactions. n.s. = *p* > 0.05

Species	PC1	PC2	PC3	Site (multi-crop → grapevine)	PC1 × site	PC2 × site	PC3 × site
*S. frugiperda*	+ 0.29, *p* < 0.001	− 0.79, *p* < 0.001	− 1.55, *p* < 0.001	n.s	n.s	+ 0.77, *p* < 0.001	+ 2.01, *p* < 0.001
*S. albula*	n.s. (*p* = 0.069)	− 0.83, *p* < 0.001	− 2.73, p < 0.001	− 3.25, *p* < 0.001	− 0.49, *p* = 0.019	n.s	n.s
*S. cosmioides*	n.s	− 0.56, *p* = 0.010	n.s. (*p* = 0.139)	+ 2.77, *p* < 0.001	n.s. (*p* = 0.092)	+ 1.08, *p* < 0.001	n.s. (*p* = 0.085)
*E. agrotina*	+ 0.56, *p* = 0.017	n.s	n.s	n.s	n.s. (*p* = 0.114)	n.s	n.s

### Temporal distribution

**Fig. 6:**
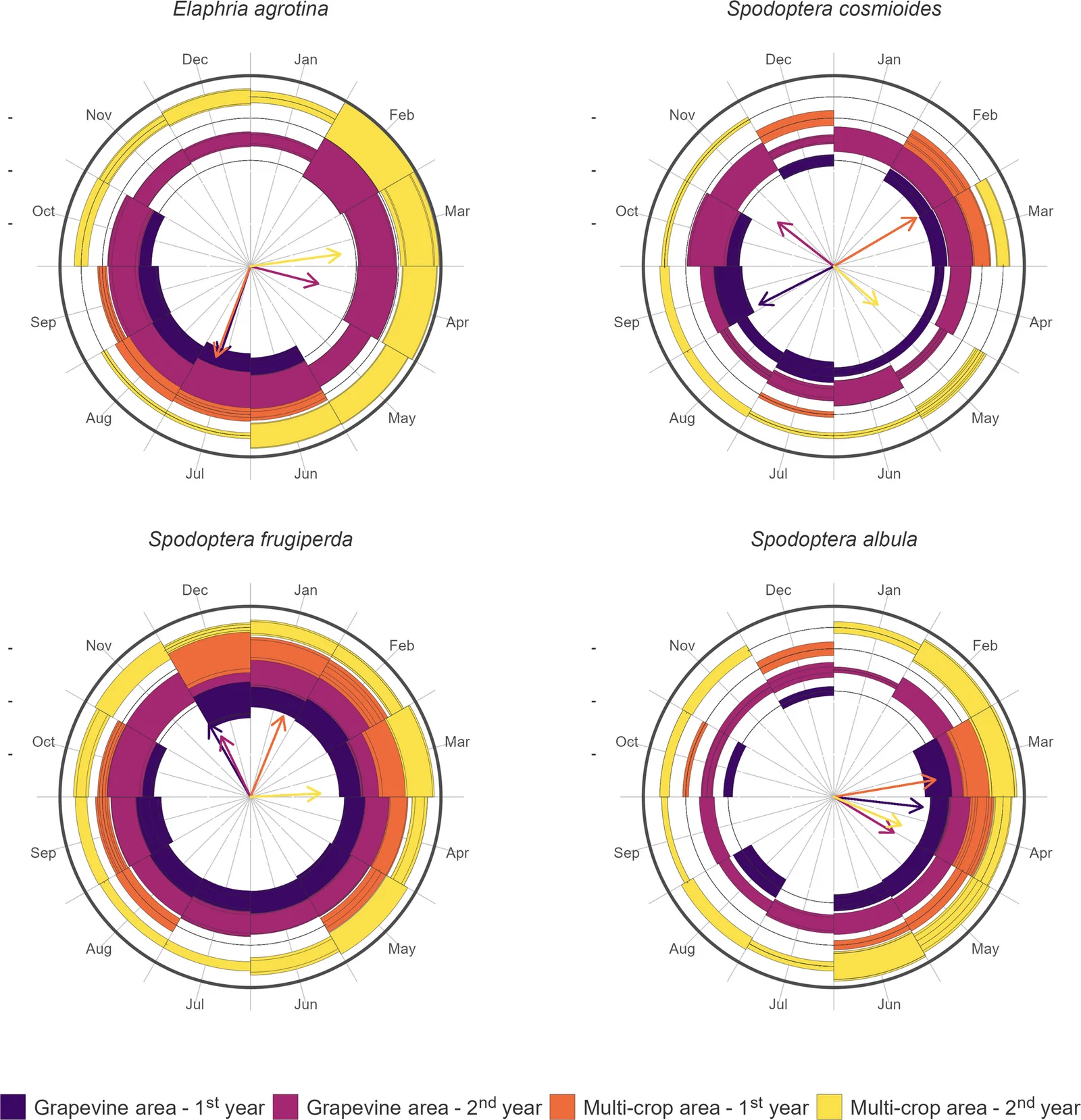
Polar panels illustrating the chronological density and population peaks of four lepidopteran species across a tropical calendar year. Concentric rings map the standardized occurrence rate of moths (log-transformed abundance) within grapevine and multi-crop areas during their 1 st and 2nd years of cultivation. Superimposed colored vectors (arrows) model the seasonal center of gravity (central estimate of the macro-peak) for each specific sub-environment. The vector angle indicates the mean temporal phase (the focal month of highest adult occurrence), while the vector length (Rayleigh’s *ρ*) demonstrates the seasonal aggregation, the intensity with which populations concentrate in that specific period rather than spreading uniformly throughout the year

Circular statistics and correlation analyses revealed clear patterns of macro-temporal structuring among the dominant taxa based on four independent annual crop cycles (Fig. 6). A negative association approaching but not reaching statistical significance was observed between *S. frugiperda* and *S. cosmioides* (*ρ* = − 0.982; *p* = 0.052), as well as between *S. frugiperda* and *S. albula* (*ρ* = − 0.988, *p* = 0.065), reflecting a trend toward seasonal temporal segregation, with their respective major peaks occurring at opposite periods of the calendar year relative to the other two congeners. Conversely, *S. albula* and *S. cosmioides* showed a positive circular association that approached, but did not reach, statistical significance (*ρ* = 0.982, *p* = 0.055). In contrast, *E. agrotina* showed negligible circular correlation with *S. frugiperda* (*ρ* = 0.067, *p* = 0.889), *S. cosmioides* (*ρ* = 0.112, *p* = 0.823), and *S. albula* (*ρ* = 0.039, *p* = 0.934). The quantitative circular metrics displayed in polar radial plots (Fig. 6) and detailed per cycle in Table S5, and pooled across cycles in Table S6 (Supplementary Information), substantiated these temporal dynamics, based on substantial sample sizes totaling 4905 specimens for *S. frugiperda*, 1143 for *S. cosmioides*, 4577 for *E. agrotina*, and 1042 for *S. albula*. Adult populations of *S. frugiperda* consistently clustered around the calendar year transition and early January across three of the four cycles, shifting toward March in the fourth (multi-crop year 2; Table S5—Supplementary Information). In marked contrast, *S. albula* exhibited a highly consistent seasonal peak restricted to late summer and early autumn across all four cycles. *Spodoptera cosmioides* peaked mainly in the second semester at the grapevine site (September–November); its much smaller multi-crop samples (*N* = 38 and 27) showed a similar or weaker seasonal signal, which was non-significant in the second year (Table S5—Supplementary Information). Meanwhile, *E. agrotina* displayed divergent mean direction vectors across sampling years, peaking in July during year 1 and in March–April during year 2, confirming that its population dynamics operate independently from the *Spodoptera* seasonal axis.

## Discussion

The local community structure includes species with wide geographic ranges and diverse larval requirements. All sampled noctuids exhibit continental distributions, combining highly polyphagous pests with species specialized in native vegetation. For example, *Bagisara repanda* Fabricius (Bagisarinae) larvae are primarily associated with native Malvaceae in disturbed areas but also occur on other botanic families, such as Anacardiaceae and Fabaceae (Wagner et al. 2011). Similarly, *Neogalea sunia* Guenée (Oncocnemidinae) ranges from the USA to Argentina (Becker 2021), despite its oligophagous larvae feeding exclusively on Verbenaceae (Bado et al. 2004). *Callopistria floridensis* (Guenée) (Eriopinae) is strictly associated with ferns (Polypodiales). Other species with oligophagous larvae belong to Noctuinae and Plusiinae, including *Xanthopastis timais* (Cramer), associated with native and ornamental Amaryllidaceae (Micheletti et al. 2017); *Mouralia tinctoides* (Guenée), restricted to Commelinaceae (Landolt 1993); and *Chloridea subflexa* (Guenée), primarily linked with *Physalis* species but also collected from other Solanaceae weeds, tomato, and chickpeas (Poole et al. 1993; Reyes et al. 2023). This combination of polyphagous pests and specialized native-feeding insects suggests the local fauna is structured by both regional biogeographic history and localized larval niches.

The main economic pests belonged to Heliothinae, Noctuinae, and Plusiinae. Within Heliothinae, notable species included *H. armigera*, introduced and established in Brazil around 2008 (Sosa-Gómez et al. 2016), and the native *Helicoverpa zea* (Boddie). Both cause major losses in soybean, maize, and cotton due to broad host plant diversity (da Silva et al. 2021; Glover et al. 2023). Within Noctuinae, representatives of the genus *Elaphria* possess polyphagous larvae that feed on seeds and dry plant tissues, as seen in *E. agrotina* and *Elaphria deltoides* (Möschler) (Specht et al. 2014). The cutworm group consisted of *Anicla infecta* (Ochsenheimer), associated mainly with Poaceae (Teston et al. 2001; Piovesan et al. 2024), and two true cutworms, *Feltia repleta* (Walker) and *Feltia subterranea* (Fabricius), which are highly abundant in tropical regions (Specht et al. 2025; Dias et al. 2025). The armyworm *Pseudaletia sequax* Franclemont is an important pest throughout the continent (Madruga et al. 2022).

Notably, *Spodoptera* species accounted for ~ 58% of all collected noctuids. *S. albula*, *S. cosmioides*, and *S. frugiperda* were represented by more than 1000 individuals, whereas *S. eridania* (Stoll) remained below 100. This composition matches findings in the Cerrado biome, which also has alternating wet and dry seasons (Piovesan et al. 2018, 2019). These *Spodoptera* species span the Americas [except *S. cosmioides*, restricted to South and Central America (Brito et al. 2024)] and exhibit relative preferences for certain families: *S. albula* with Fabaceae (Montezano et al. 2013a, b); *S. cosmioides* with Asteraceae and Solanaceae (Specht and Roque-Specht 2016); *S. eridania* with Asteraceae (Montezano et al. 2014a, b); and *S. frugiperda* with Poaceae (Montezano et al. 2018). Within Plusiinae, loopers such as *C. includens*, *Autoplusia egena* (Guenée), and *Ctenoplusia oxygramma* (Geyer) are polyphagous pests associated with major crops such as soybeans, beans, and vegetables (Specht et al. 2007, 2015; Santos et al. 2021).

The contrasting patterns between the two sites are better explained by landscape connectivity and habitat stability shaping overall abundance and assemblage composition, rather than by an overall difference in diversity or richness in favor of either site once sampling effort is accounted for (Table 1). The much larger number of individuals captured at the grapevine likely stems from its connectivity with the surrounding Caatinga biome, which facilitates species spillover into the agricultural matrix and enhances the pool of individuals available for capture (Novotný et al. 2015; Taranto et al. 2023), together with the structural stability of a perennial, continuously managed crop. Perennial vines and spontaneous weed cover offer a stable environment that can maintain large populations of a few well-adapted species even where overall crop diversity at the site is low (Bosco et al. 2023; Seymour et al. 2020; Tassoni et al. 2024). Once this effort/abundance advantage is accounted for, however, per-individual species richness was in fact higher at the more heterogeneous multi-crop station, indicating that habitat stability and species richness respond to different aspects of landscape structure and should not be conflated (Gotelli and Colwell 2001).

This landscape heterogeneity may contribute to structural shifts within the *Spodoptera* complex (Parra et al. 2022). For instance, *S. cosmioides* was six times more frequent in the grapevine area. This is probably associated with the widespread presence of *Amaranthus deflexus* L., a weed common in São Francisco Valley vineyards (Lessa et al. 2021), as the genus *Amaranthus* is a highly favorable host plant for *S. cosmioides* (Páez Jerez et al. 2022). Additionally, grapevine itself may serve as a direct host: the Embrapa Semiarid Insect Collection holds records of *S. cosmioides* larvae feeding on its leaves in the region. Similarly, *S. albula*, the third most frequent species in the multi-crop area, uses common regional weeds like *Portulaca oleracea* L. as alternative host reservoirs (Montezano et al. 2013a, b; Specht and Roque-Specht 2016).

Contrary to what the raw capture totals suggest, once we accounted for sampling effort, the multi-crop experimental station showed higher species richness per individual captured than the grapevine site (Table 1; Table S2—Supplementary Information). This is consistent with the greater diversity of crops and associated non-crop vegetation present within the 400-m radius of the multi-crop trap, which may provide a wider range of larval host plants and adult microhabitats per unit of sampling effort than the more structurally uniform vineyard (Sirami et al. 2019). The frequent anthropogenic interventions and simplified plot margins typical of an experimental station (Blumgart et al. 2023) may still explain the comparatively low overall abundance recorded there, without implying lower richness once that lower abundance is accounted for; high spatial turnover among Caatinga insect communities (Arnan et al. 2018) may in fact be compatible with a smaller, more heterogeneous plot accumulating a broad species pool relative to its capture volume. This suggests that the vineyard’s connectivity with Caatinga remnants inflates the number of individuals captured, and hence its raw richness and diversity advantage in Fig. 2, predominantly through elevated abundance of a limited number of well-adapted species (e.g., *E. agrotina*, *S. frugiperda*), rather than through a broader underlying species pool.

Wind speed and precipitation jointly shaped capture rates through PC2, the wind-precipitation axis (Fig. 4), reflecting the strong covariation between these two variables at the monthly scale in this seasonally dry system. *S. frugiperda* and *S. albula* were consistently more abundant at lower wind speed and higher precipitation, in both the concurrent and preceding months; *S. cosmioides* showed the same pattern only in the preceding month, while *E. agrotina* showed no detectable response to this axis in either month (Tables 2 and 3). This pattern is consistent with two well-established, non-exclusive mechanisms. First, wind intensity is a direct physical constraint on flight and trap efficiency: light-trap catches of Noctuidae and related moths drop as wind speeds increase, both through reduced mechanical capture and through active suppression of takeoff and disorientation at the light source (Douthwaite 1978; McGeachie 1989). Second, rainfall is a resource-driven demographic driver; the onset of the rainy season triggers a pulse in plant biomass, flushing new foliage that transforms a resource-poor landscape into a highly productive habitat (Vasconcellos et al. 2010; Fischer et al. 2022). While nightly precipitation can temporarily depress short-term flight activity and reduce immediate trap efficiency (Holyoak et al. 1997), timely seasonal rainfall overrides these constraints. This demographic mechanism is evident in polyphagous groups such as *Spodoptera*, where early-season rain events trigger population outbreaks and mass flights (Sharma et al. 2002). Because wind and rainfall covary strongly at the monthly scale in this seasonally dry system, the present data cannot statistically separate a sampling-efficiency artifact of wind from a genuine demographic response to rainfall; distinguishing the two would require nightly, rather than monthly, climatic data.

A third climatic axis dominated by solar radiation, with a smaller, opposite-signed contribution from precipitation (PC3; Table S3, Table S4—Supplementary Information) was also important, though its influence differed by species and by time window. *S. albula* was more abundant under higher solar radiation in both the concurrent and preceding month, *E. agrotina* only in the concurrent month, and *S. frugiperda* only in the preceding month (Tables 2 and 3), consistent with reports that reduced cloud cover and greater sky brightness increase Noctuidae light-trap catches, most plausibly by increasing the effective attraction radius of the light source (Yela and Holyoak 1997). On the thermal–humidity axis (PC1), *E. agrotin*a and *S. frugiperda* abundance increased with higher temperature in the preceding month, consistent with warmer temperatures accelerating metabolic rates and larval development (Du Plessis et al. 2020), increasing adult emergence and trap catches weeks later (Moreno et al. 2021). *S. cosmioides*, by contrast, showed no response, and was instead more strongly predicted by site than by any climatic axis on its own (Table 3). Several species’ climatic responses also differed between the two sites (Tables 2 and 3), consistent with evidence that landscape and habitat context modulate, rather than simply add to, the effect of climate on arthropod population dynamics in agricultural systems (Bloom et al. 2020; Outhwaite et al. 2022). This reinforces the broader interpretation above that landscape connectivity and habitat stability, not climate alone, shape the observed differences between the grapevine and multi-crop assemblages. The dominance of *E. agrotina* in the grapevine area, where it was the most frequent species, reflects an ecological strategy that differs from that of the leaf-feeding *Spodoptera* complex. This study represents the first report of *E. agrotina* in the Brazilian semi-arid region, a species otherwise widely distributed across the Americas (Kimball 1965; Pastrana 2004). Although associated with crops such as soybean, cotton, sugarcane, and maize (Specht et al. 2014; Nascimento et al. 2024), none of these was present at the study sites. Instead, its success is explained by feeding habits: caterpillars of the genus *Elaphria* prefer dry food sources, such as dried kernels, ear bases, and necrotic leaves (Specht et al. 2014). Staggered table grape production on the studied farm meant all phenological stages were present simultaneously, leading to continuous pruning that left stalks and leaves on the soil. This management practice provided a constant supply of dried food resources tailored for *E. agrotina*. Additionally, the native flora of the adjacent Caatinga, highly adapted to water scarcity through succulent plants and terrestrial bromeliads (Rocha et al. 2004), likely serves as an alternative, resilient resource reservoir.

The observed trend toward seasonal segregation between *S. frugiperda* and *S. cosmioides* likely reflects distinct host preferences and differences in net reproductive rates, mean generation times, and biotic potential (Montezano et al. 2019; Specht and Roque-Specht 2019). Although environmental analyses indicate that both pests benefit from similar microclimatic triggers, such as rainfall, the temporal ordering of their macro-population peaks may reduce interspecific competitive overlap. This temporal ordering allows them to exploit the same fruit-growing agroecosystems without causing population collapses through direct nutritional niche overlap within the same month. Additionally, the asynchronous behavior of *E. agrotina* confirms that it occupies a separate detritivorous niche at the margins of the temporal web.

In general, the noctuid community associated with this area, characterized by high dispersal capacity and fecundity, is well adapted to exploit the erratic availability of host plants during the short rainy periods typical of the semi-arid region. No endemic species were detected, likely because the region has undergone a major conversion to irrigated fruit farming since the 1970 s, involving extensive use of phytosanitary products (Ferreira et al. 2014). Notably, among all sampled species, only representatives of Heliothinae have records of remaining in pupal diapauses in tropical areas (Specht et al. 2025) or engaging in long-distance migratory movements (He et al. 2024; Paula-Moraes et al. 2024). The other species of agricultural importance exhibit high dispersal capacity and are largely migratory, such as *A. infecta*, *C. includens*, *C. oxygramma*, *S. eridania*, and *S. frugiperda* (Nagoshi et al. 2023).

This study provides novel insights into the biodiversity and ecological drivers of Noctuidae communities in an underexplored dryland fruit-producing landscape. These findings should nonetheless be interpreted in light of the study’s design: each site was monitored with a single trap, so habitat and site effects cannot be fully disentangled from other unmeasured spatial differences between locations. Future work should experimentally test the climatic and host-plant hypotheses proposed here by collecting immatures for host identification and evaluating landscape connectivity using more direct approaches, such as population genetics, to better understand and promote an optimized foundation for integrated pest management strategies in the region.

## Supplementary Information

Below is the link to the electronic supplementary material.

ESM 1 DOCX (1.61 MB)

## Data Availability

The raw datasets and R scripts used to perform the statistical analyses in the current study are available from the corresponding author upon reasonable request.
