# Supplementary material for: Landscape connectivity and climatic drivers shaping Noctuidae communities in a Brazilian seasonally dryland fruit-producing region
Source: Neotrop Entomol. 2026 Oct 28;55(1):96. doi: 10.1007/s13744-026-01446-y (PMC13719849; doi:10.1007/s13744-026-01446-y)
Supplement: Supplementary file 1 [file 13744_2026_1446_MOESM1_ESM.docx]

**Table S1** Specific abundance (n) of Noctuidae moths collected with light traps (July 2015 - June 2017) in a multi-crop and grapevine area in a seasonally dryland fruit-producing region, São Francisco Valley, Brazil.

| Species/Subfamily | Multi-crop area | Grapevine area | Total |
| --- | --- | --- | --- |
| Bagisarinae |  |  |  |
| *Bagisara repanda* (Fabricius) | 62 | 86 | 148 |
| Eriopinae |  |  |  |
| *Callopistria floridensis* (Guenée) | 0 | 2 | 2 |
| Heliothinae |  |  |  |
| *Helicoverpa armigera* (Hübner) | 1 | 34 | 35 |
| *Helicoverpa zea* (Boddie) | 4 | 4 | 8 |
| *Chloridea subflexa* (Guenée) | 12 | 32 | 44 |
| Noctuinae |  |  |  |
| *Elaphria agrotina* (Guenée) | 1,040 | 3,537 | 4,577 |
| *Elaphria deltoides* (Möschler) | 1 | 0 | 1 |
| *Elaphria jalapensis* (Schaus) | 58 | 4 | 62 |
| *Elaphria subobliqua* (Walker) | 2 | 1 | 3 |
| *Elaphria villicosta* (Walker) | 4 | 0 | 4 |
| *Feltia repleta* (Walker) | 4 | 4 | 8 |
| *Feltia subterranea* (Fabricius) | 23 | 247 | 270 |
| *Anicla infecta* (Ochsenheimer) | 2 | 9 | 11 |
| *Pseudaletia sequax* Franclemont | 0 | 18 | 18 |
| *Spodoptera albula* (Walker) | 617 | 425 | 1,042 |
| *Spodoptera cosmioides* (Walker) | 65 | 1,078 | 1,143 |
| *Spodoptera eridania* (Stoll) | 18 | 62 | 80 |
| *Spodoptera frugiperda* (Smith) | 1,471 | 3,434 | 4,905 |
| *Xanthopastis timais* (Cramer) | 0 | 1 | 1 |
| Oncocnemidinae |  |  |  |
| *Neogalea sunia* (Guenée) | 5 | 1 | 6 |
| Plusiinae |  |  |  |
| *Chrysodeixis includens* (Walker) | 9 | 26 | 35 |
| *Autoplusia egena* (Guenée) | 1 | 0 | 1 |
| *Mouralia tinctoides* (Guenée) | 0 | 1 | 1 |
| *Ctenoplusia oxygramma* (Geyer) | 0 | 2 | 2 |
| Total | 3,399 | 9,008 | 12,407 |

**Table S2** Species richness, Shannon diversity, and Pielou's evenness for the multi-crop and grapevine sites, comparing raw (full-effort) values with individual-based rarefaction/Chao1 estimates standardized to equal sampling effort. São Francisco Valley, Brazil.

| **Metric** | **Multi-crop (observed)** | **Grapevine (observed)** | **Grapevine, rarefied to N = 3,399** |
| --- | --- | --- | --- |
| Individuals (N) | 3,399 | 9,008 | 3,399 (by design) |
| Species richness (S) | 19 | 21 | 17.3 (SE 1.35) |
| Chao1 ± SE | 20.0 ± 1.81 | 23.0 ± 2.87 | — |
| Shannon H' | 1.399 | 1.405 (full effort) | 1.405 (95% CI 1.37–1.43) |
| Pielou J' | 0.475 | 0.462 (full effort) | 0.493 (95% CI 0.47–0.52) |

**Table S3** Loadings of the ten standardized concurrent-month climatic variables on the first three principal components (cumulative 91.5% of variance; n = 48 occasions). São Francisco Valley, Brazil.

| **Variable** | **PC1** | **PC2** | **PC3** |
| --- | --- | --- | --- |
| Mean temperature | 0.35 | -0.31 | 0.03 |
| Max temperature | 0.38 | -0.20 | 0.10 |
| Min temperature | 0.30 | -0.39 | 0.13 |
| Mean relative humidity | -0.37 | -0.22 | -0.19 |
| Max relative humidity | -0.39 | -0.08 | -0.20 |
| Min relative humidity | -0.34 | -0.32 | -0.15 |
| Solar radiation | 0.25 | -0.23 | -0.76 |
| Wind speed | 0.08 | 0.57 | 0.04 |
| Precipitation | -0.17 | -0.41 | 0.54 |
| ET0 | 0.38 | 0.06 | -0.04 |
| % variance explained | 59.1% | 25.0% | 7.4% |

**Table S4** Loadings of the ten standardized one-month-lagged (lag-1) climatic variables on the first three principal components (cumulative 91.7% of variance; n = 46 occasions). São Francisco Valley, Brazil.

| **Variable** | **PC1** | **PC2** | **PC3** |
| --- | --- | --- | --- |
| Mean temperature | 0.34 | -0.34 | 0.05 |
| Max temperature | 0.38 | -0.22 | 0.12 |
| Min temperature | 0.28 | -0.41 | 0.17 |
| Mean relative humidity | -0.37 | -0.24 | -0.17 |
| Max relative humidity | -0.39 | -0.07 | -0.22 |
| Min relative humidity | -0.34 | -0.32 | -0.14 |
| Solar radiation | 0.24 | -0.25 | -0.78 |
| Wind speed | 0.11 | 0.55 | 0.03 |
| Precipitation | -0.19 | -0.38 | 0.50 |
| ET0 | 0.39 | 0.05 | -0.07 |
| % variance explained | 58.0% | 26.2% | 7.6% |

**Table S5** Per-cycle (site × harvest-year) circular statistics for the four dominant Noctuidae species in two areas (grapevine and multi-crop), São Francisco Valley, Brazil: sample size (N), mean direction (μ, converted to an approximate calendar date), bootstrap 95% confidence interval, mean resultant length (ρ), and Rayleigh test p-value. n.s. = not significant (p > 0.05).

| **Species** | **Cycle** | **N** | **Mean vector μ**  **(approx. date)** | **95% CI** | **ρ** | **Rayleigh p** |
| --- | --- | --- | --- | --- | --- | --- |
| *S. frugiperda* | Grapevine, Y1 | 2,081 | 343.92° (~Dec 13) | [341.59°, 346.24°] | 0.649 | < 0.0001 |
| *S. frugiperda* | Grapevine, Y2 | 1,353 | 348.27° (~Dec 18) | [342.95°, 353.60°] | 0.389 | < 0.0001 |
| *S. frugiperda* | Multi-crop, Y1 | 1,185 | 7.73° (~Jan 07) | [5.00°, 10.46°] | 0.704 | < 0.0001 |
| *S. frugiperda* | Multi-crop, Y2 | 286 | 78.76° (~Mar 20) | [68.45°, 89.07°] | 0.433 | < 0.0001 |
| *S. albula* | Grapevine, Y1 | 164 | 88.96° (~Mar 30) | [82.21°, 95.70°] | 0.740 | < 0.0001 |
| *S. albula* | Grapevine, Y2 | 261 | 115.48° (~Apr 26) | [104.69°, 126.27°] | 0.433 | < 0.0001 |
| *S. albula* | Multi-crop, Y1 | 310 | 71.41° (~Mar 12) | [69.50°, 73.32°] | 0.956 | < 0.0001 |
| *S. albula* | Multi-crop, Y2 | 307 | 106.73° (~Apr 17) | [97.95°, 115.52°] | 0.483 | < 0.0001 |
| *S. cosmioides* | Grapevine, Y1 | 217 | 248.56° (~Sep 08) | [241.38°, 255.73°] | 0.651 | < 0.0001 |
| *S. cosmioides* | Grapevine, Y2 | 861 | 319.78° (~Nov 19) | [314.11°, 325.44°] | 0.451 | < 0.0001 |
| *S. cosmioides* | Multi-crop, Y1 | 38 | 49.11° (~Feb 18) | [38.67°, 58.54°] | 0.832 | 1.5×10⁻¹¹ |
| *S. cosmioides* | Multi-crop, Y2 | 27 | 126.94° (~May 08) | [65.28°, 213.11°] | 0.252 | 0.182(n.s.) |
| *E. agrotina* | Grapevine, Y1 | 399 | 200.68° (~Jul 22) | [197.26°, 204.09°] | 0.829 | < 0.0001 |
| *E. agrotina* | Grapevine, Y2 | 3,138 | 97.49° (~Apr 08) | [94.40°, 100.59°] | 0.434 | < 0.0001 |
| *E. agrotina* | Multi-crop, Y1 | 26 | 202.60° (~Jul 24) | [190.42°, 214.79°] | 0.857 | 7.8×10⁻⁹ |
| *E. agrotina* | Multi-crop, Y2 | 1,014 | 73.45° (~Mar 15) | [70.89°, 76.01°] | 0.764 | < 0.0001 |

**Table S6** Sample size, mean date of seasonal peak occurrence with 95% bootstrap confidence interval, mean vector concentration (Rayleigh ρ), and Rayleigh test for non-uniformity (seasonality) for each of the four dominant species. São Francisco Valley, Brazil.

| **Species** | **N individuals** | **Mean peak date** | **95% CI (bootstrap)** | **Rayleigh ρ** | **Rayleigh test** |
| --- | --- | --- | --- | --- | --- |
| *S. frugiperda* | 4,905 | ~25 Dec (day 360) | 23–28 Dec | 0.544 | p < 0.001 |
| *E. agrotina* | 4,577 | ~9 Apr (day 100) | 7–13 Apr | 0.435 | p < 0.001 |
| *S. cosmioides* | 1,143 | ~7 Nov (day 311) | 1–13 Nov | 0.384 | p < 0.001 |
| *S. albula* | 1,042 | ~1 Apr (day 91) | 29 Mar–4 Apr | 0.622 | p < 0.001 |
